# A randomised comparison of UK genetic risk counselling services for familial cancer: psychosocial outcomes

**DOI:** 10.1038/sj.bjc.6602081

**Published:** 2004-08-10

**Authors:** P Hopwood, D Wonderling, M Watson, A Cull, F Douglas, T Cole, D Eccles, J Gray, V Murday, M Steel, J Burn, K McPherson

**Affiliations:** 1 Christie Hospital NHS Trust, The CRC Psychological Medicine Group, Stanley House, Wilmslow Road, Withington, Manchester, M20 4BX, UK; 2 Cancer and Public Health Unit & Health Services Research Unit, London School of Hygiene and Tropical Medicine, Keppel Street, London, WC1E 7HT, UK; 3 Royal Marsden Hospital and Psychology Research Group & Section of Cancer Genetics, Institute of Cancer Research, Downs Road, Sutton, Surrey, SM2 5PT, UK; 4 Cancer Research UK Edinburgh Oncology Unit, Western General Hospital, Crewe Road South, Edinburgh, EH4 2XR, UK; 5 Institute of Human Genetics, International Centre for Life, Central Parkway, Newcastle upon Tyne, NE1 3BZ, UK; 6 Department of Medical Genetics, Women's Hospital, Edgbaston, Birmingham, UK; 7 Wessex Clinical Genetics Service, Level G, Princess Ann Hospital, Coxford Road, Southampton, SO16 5YA, UK; 8 Institute of Medical Genetics, University Hospital of Wales, Heath Park, Cardiff, CF4 4XN, UK; 9 Department of Medical Genetics, St George's Hospital, London, UK; 10 Tayside Regional Clinical Genetics Service, Ninewells Hospital & Medical School, Dundee, DD1 9SY, UK; 11 Nuffield Department of Obstetrics and Gynaecology, Research Institute, Churchill Hospital, Oxford OX3 7LJ, UK

**Keywords:** breast cancer family history, risk counselling, psychological distress, satisfaction

## Abstract

The aim of the study was to compare psychosocial outcomes for 50 new clinic attendees, referred for cancer genetic counselling to five UK centres. The centres represented England, Scotland and Wales, and were randomly selected from groups ranked by different levels of clinical activity in cancer genetics practice. Questionnaires assessed demographic data, risk perception, mental health and use of health services pre-consultation and at 1 and 12 months follow-up. Satisfaction was measured for attendees and referring doctors at follow-up. A total of 256 unaffected adults fulfilled the study criteria. The five centres varied widely with respect to service organisation and activity, but all had a greater proportion of unaffected attendees with a breast cancer risk (61–91%) than either a bowel cancer risk (0–33%) or ovarian cancer risk (3–25%). There were no significant differences in the psychosocial data between centres pre-counselling. No significant change over time occurred for any of the centres for risk perception or general psychological distress. There were significant differences between centres in reduction of cancer worry from baseline to 12 months and with the number of women who were recommended to have mammographic surveillance who had not received this. Overall, one-third of women for whom mammography had been recommended had not been screened within 1 year of follow-up. Subsequent attendance at the GP, but not at a hospital, was associated with risk level, but differences between centres could not be analysed. Satisfaction differed significantly between centres for 4 : 14 aspects of service provision and with 3 : 17 items concerning communication; satisfaction was high overall. Over 90% of referring doctors were moderately/very satisfied with the service, but 23% were dissatisfied with waiting times and 19% with access to preventive treatment. Results differed significantly between centres for doctor's satisfaction with the provision of referral criteria and prescribing information. In conclusion, there were relatively few significant differences in psychosocial outcomes between centres, considering the wide variation in service organisation and activity. These significant differences were not consistent across the centres, therefore, differences could not be linked to specific aspects of service provision.

Most NHS regionally based genetics centres now provide clinics that deal specifically with risk assessment for familial cancers, such as breast and colon cancers, in response to an increased demand for information about cancer risks, for cancer screening and advice on risk management. These cancer genetics services (CGSs) have evolved in an *ad hoc* fashion according to local expertise and resources. In phase one of this research (Wonderling *et al*, 2001), the clinical activity of 22 of the 24 regional CGSs was evaluated over the same 1-month period, in order to describe the service provision nationally and make regional comparisons. We found that all cancer genetics clinics were dominated by referrals from families with a history of breast cancer and there were significant variations between the regions, for example in organisation of clinics, levels of staffing, number of referrals per million population, waiting time for first appointment and risk level of families referred, and the results implied that there were substantial variations in access to molecular genetic testing and to cancer screening. The proportion of referrals from primary care ranged from 29 to 70%, with similar wide variations reported recently for South East Scotland (Campbell *et al*, 2003).

It is important to know whether these service variations resulted in different psychosocial outcomes for service users with respect to their risk knowledge, mental health or health care behaviour. Phase two of our study, presented here, was designed to address this question. If there were significant differences, these could have cost consequences and implications for the optimal use of available NHS resources, as well as for the further development of regional services. Prior to this study, psychosocial research had focused on individual centres, evaluating the impact of risk counselling on psychological status and risk perception in women at increased risk of breast cancer. A meta-analysis of 12 of these studies (Meiser and Halliday, 2002) has now confirmed a reduction in psychological distress after genetic risk counselling, although the combined results did not quite reach statistical significance. There was a significant decrease in short-term anxiety and a significant improvement in the accuracy of women's perception of their personal risk. While the benefit of anxiety reduction was short lived, there was no evidence of an increase in general psychological distress in studies with a longer follow-up, irrespective of whether counsellees initially underestimated or overestimated their risk. Therefore, there appears to be a beneficial effect of risk counselling on the perceived risk accuracy without psychological detriment. Meiser *et al* commented that it was not possible to include other outcomes of risk counselling, such as mammographic screening uptake, because of an insufficiency of studies; findings to date have been inconsistent (Kash *et al*, 1995; Audrain *et al*, 1999; Schwartz *et al*, 1999).

The benefit from risk counselling was further confirmed in a recent systematic review of the psychosocial literature (Butow *et al*, 2003), based on two randomised controlled trials and seven longitudinal studies. However, the strength of the conclusions was considered to be limited by the lack of randomised study designs, a problem addressed by the current research.

In the first study to compare the psychosocial outcomes of different forms of service provision, Brain *et al* (2000) evaluated the referred women who were randomly allocated to one of two different service models for familial breast cancer within Wales. Women who received risk counselling from a multidisciplinary genetics team had a significantly greater improvement in risk knowledge than those allocated to the surgical service, but this was achieved with an increased service cost. No differences in mental health or women's satisfaction with the respective CGSs were found; women's anxiety and cancer worry decreased over 9 months following risk counselling in both arms of the trial. In the most recent evaluation in South East Scotland, Fry *et al* (2003) found no significant differences in psychosocial outcomes for women referred to existing regional genetics services or a novel community-based service, within a randomised trial. It is important to know if these findings hold true for other UK centres that differ from each other in size, organisation and demands on service delivery.

In general, a deficiency in many psychosocial studies to date has been the lack of systematic reporting of details of the specific CGS and of the clinic population. This issue is addressed in this study.

The aims of this phase 2 study were three-fold. First, in each of five centres, to record the changes in risk perception, psychological distress, health care behaviour and use of health care resources in the year following cancer genetic counselling for families with a history of cancer. Second, to ascertain satisfaction with the service of both service users and their referring doctors (GPs and hospital clinicians), and third, to describe regional variations in outcomes that could be attributable to service organisation or activity.

## METHODS

### Selection of centres

During phase 1 of the study, 22 of a possible 24 regional CGSs were recruited to record all of their genetic counselling activity over a pre-specified 4-week period. For the phase 2 studies, 20 centres (with full data available at the time of randomisation) were ranked according to the rate of referrals received per million population, as an indicator of service activity, and formed into five sequential groups. One centre was then randomly selected from each of these groups, and that centre asked to participate. By chance, the five CGSs were located in regions in England, Scotland and Wales.

### Sample

Each CGS was required to recruit 50 individuals newly attending for cancer genetic risk counselling. Individuals were excluded from participation in the study if they had already been diagnosed with cancer at any time or if they were under the age of 18. A nominated member of the CGS staff recruited participants prior to their first genetic counselling consultation. After reading an information sheet and having an opportunity to ask questions about the study, participants gave their written consent. They completed a questionnaire booklet immediately prior to their first consultation and the CGS clinician completed a study report form following the consultation. Participants were sent follow-up questionnaires by post 1 and 12 months after the initial consultation. At approximately 6 months after recruitment, the doctor (GP or clinician) who had referred the individual to the CGS was sent a questionnaire to ascertain satisfaction with the service provided. Response from participating service users and from their referring doctors was encouraged by telephone reminders after 2 weeks and written reminders 2 weeks later. Ethical approval for all aspects of the study was obtained through the Multicentre Research Ethics Committee.

### Psychosocial measures

Psychosocial outcomes were assessed using patient self-report questionnaires; where available, standardised measures were used. Protocol-specific scales were derived from measures used in other cancer genetics studies (Hopwood *et al*, 1998; Watson *et al*, 1998). The data collected included the following:

#### Demographic information

Patients were asked to indicate their age, gender, ethnic background, level of education and occupation.

#### Genetic risk evaluation

Perceived cancer risk was assessed using a protocol-specific scale by asking subjects to rate their chances of developing cancer compared to the general population, using five response categories (very much lower–very much higher).

#### Psychological distress

General mental health was assessed using the 28-item General Health Questionnaire (GHQ) (Goldberg and Williams, 1988), a self-report measure for detecting psychiatric disorder in nonpsychiatry settings.

#### Cancer-specific distress

Cancer worry, an indicator of cancer-specific distress, was assessed using the modified Cancer Worry Scale (CWS) (Watson *et al*, 1998; Hopwood *et al*, 2001), originally developed by Lerman *et al* (1994). The six-item scale has been shown to have good reliability (Brain *et al*, 2000; Hopwood *et al*, 2001), but no clinical case cutoff score is derived.

### Health care behaviour and use of health care resources

Health care behaviour was measured using protocol-specific questions to ascertain the following: use of cancer screening tests (e.g. mammography, colonoscopy, ovarian screening), breast self-examination, clinical breast examination, attendance at the GP surgery, well-woman check-ups, private health check-ups and other hospital visits. Subjects were asked to indicate their attendances for health care in the 6 months prior to the genetics consultation (assessed at baseline), in the month following the visit and in the 12 months following the visit.

### Satisfaction with the CGS

Satisfaction with the service provided was assessed by counsellees using a protocol-specific 14-item scale. Subjects were asked to indicate their satisfaction on a four-point scale (not at all–very satisfied).

### Satisfaction with communication (Fallowfield, 1998)

Satisfaction with patient–clinician communication and key aspects of the consultation was measured using a previously tested 17-item scale (Fallowfield, 1998). Respondents were asked to indicate their level of agreement (or disagreement) with positive and negative statements about the consultation on a five-point item response scale.

### Satisfaction of referring doctors

Referring GPs and hospital clinicians were asked to indicate their satisfaction with nine aspects of service provision, both in terms of their overall impression of the local service and in respect of the individual study participant they had referred.

### Scoring and statistical analysis

The GHQ-28 was scored using the preferred binary (0–0, 1–1) system giving an overall score on a scale from zero to 28, with higher scores representing higher distress. A cutoff score of ⩾10 was used to indicate those individuals who were psychologically distressed (after Hopwood *et al*, 1998). Item responses on the CWS were scored 1–4 for each question, giving a summary score of 6–24; higher scores represented higher levels of worry. Satisfaction with patient–doctor communication was evaluated using five-point responses, scored 0–4, giving an overall score between zero and 68, with higher scores representing higher satisfaction. Risk accuracy was assessed by comparing perceived risks with clinician assessments within three broad categories: population risk or marginally increased, sufficient for screening (moderate risk) or family history indicative of possible autosomal dominant mutation (high risk). Data were used to create the following subgroups: accurate, overestimates or underestimates.

The study was designed with the intention of being descriptive and hypothesis generating, and hence it was not powered to detect differences between regions. However, statistical analyses were conducted to see if the variability observed between regions could have occurred by chance. The Kruskal–Wallis test was used for waiting time and other quantitative variables. The Wilcoxon test and McNemar's test were used to analyse change over time in quantitative and categorical data, respectively. The results presented in this paper relate to the sample of patients who responded to the pre-counsel and both follow-up questionnaires.

## RESULTS

### Participating centres

The characteristics of the five participating centres with respect to service activity are shown in Table 1

**Table 1:** Characteristics of participating cancer genetics services (CGSs)

	**Centre A**	**Centre B**	**Centre C**	**Centre D**	**Centre E**	**All five CGSs**
*Regional data*						
Population (m)	2.9	5.2	3.1	2.2	0.5	13.9
Referral rate (per million pop. p.a.)	130	208	268	298	353	225
No. of designated clinics for cancer risk referrals (and location)	0	2 (central hospitals)	6 (breast and cancer clinics, local hospitals and breast screening unit)	10 (general hospitals)	1 (breast screening unit)	19
No. of nondesignated clinics for cancer risk referrals (and location)	7 (medical genetics clinics)	>22 across different clinic sites	0	0	2 (medical genetics clinics)	>31
						
*Service provision*						
Extent of genetic testing (% of elevated risk)	25	31	26	32	18	26
Referral criteria (% sent questionnaire)	6	89	39	88	70	59
Mammography provided directly by CGS	No	No	Yes (some clinics)	No	Yes (some clinics)	—
Median waiting time	19	23	29	26	31	24
% subjects seen in designated CGS clinic	94	42	75	100	100	82
						
*Staffing*						
Consultant (WTE)	1.0	1.0	0.7	1.0	0.4	4.1
Other medic. (WTE)	0.2	0.2	1.1	0.2	0.2	1.9
Genetic counsellor (WTE)	2.3	1.5	2.3	1.0	0.1	7.2
Dedicated cancer genetics consultant	Yes	No	No	Yes	No	—
% Subjects seen by CGS consultant	53	58	56	76	100	70
% Subjects seen by CGS clinician	100	75	100	93	100	94

. Together, they served a population of 13.9 million (range 0.5–5.2 m) with a variation in referral rate from 130 to 353 per million population per annum, although these rates were not consequent on catchment size. The mean number of sites for cancer genetics clinics per service was 10, with a range from three to over 25 clinics in a range of locations. Centre A had no designated cancer genetics clinics, while centres C and D provided designated clinics at a total of 16 different hospitals. The other two centres (B and E) provided both designated CGSs and cancer genetics consultations within general medical genetics clinics. Clinics were held in a variety of hospital departments or screening units, and breast clinics. Across the regions, clinic frequency ranged between two per week and one per 2–3 months, according to local demand and service organisation. While other risk assessment services were thought to exist in each region, three of the five centres had no precise knowledge of these and two reported some awareness; centre A specified the Breast Screening Unit and centre D specified the Breast Screening Unit, Neurogenetics clinics and Dermatogenetics clinics. Staffing level/type/grade, clinic type, waiting time and screening provision also varied, consistent with findings for UK services as a whole (Wonderling *et al*, 2001). The proportion of counsellees seen in different locations (central clinic *vs* outreach) also varied widely. Overall, general practitioners made 67% of referrals and specialists (predominantly breast surgeons and gynaecologists) accounted for 34%. GP referrals varied significantly from between 52% (Centre A) to 95% (Centre E), but there was little difference in the reported reason for referral, with 89% or more of doctors specifying risk assessment as the main reason. Both referral routes resulted in a waiting time for appointment of up to 6 months for the majority of referrals.

### Sample characteristics

A total of 271 individuals consented to participate, of whom 256 fulfilled protocol criteria pre-counsel (range 49–52 by centre). Reasons for exclusion after consent included cancer diagnosis (11) and missing baseline questionnaire (4). Concurrent research studies in two centres interfered with the plan to recruit consecutive attendees and additional time was needed to accrue the study sample in three centres. Thus, the sample can best be described as a sample of convenience. Socio-demographic data (shown in Table 2

**Table 2:** Sample characteristics

	**Centre A**	**Centre B**	**Centre C**	**Centre D**	**Centre E**	**All five CGSs**
*Sample response*						
Pre-counsel	52 (100%)	52 (100%)	49 (100%)	52 (100%)	51 (100%)	256 (100%)
1 month	45 (86%)	45 (86%)	48 (98%)	48 (92%)	48 (94%)	234 (91%)
12 months	40 (77%)	37 (71%)	39 (80%)	43 (83%)	43 (84%)	202 (79%)
All three	36 (69%)	36 (69%)	39 (80%)	41 (79%)	40 (78%)	192 (75%)
						
*Sociodemographics*						
Gender (% male)	6%	17%	0%	7%	0%	6%
Mean age (years), range	40 (24, 63)	41 (24, 72)	42 (23, 68)	43 (22, 67)	40 (23, 61)	41 (22, 72)
Ethnic minority	0	1 (3%)	3 (8%)	0	0	4 (2%)
University educated	61%	42%	62%	34%	35%	46%
Professional or managerial	50%	26%	53%	48%	31%	42%
						
*Site of familial cancer*						
Breast	69%	61%	74%	71%	98%	75%
Bowel	6%	33%	23%	24%	0	17%
Ovary	25%	6%	8%	7%	3%	9%
Other	0	3%	3%	5%	0	2%
						
*Risk level (clinician estimate)*						
Population-level risk	17%	14%	18%	20%	26%	19%
Moderate risk	35%	56%	50%	63%	69%	56%
High risk	48%	31%	32%	26%	5%	25%

) revealed the same preponderance of women from breast cancer families and lack of ethnic mix as described in the national study (Wonderling *et al*, 2001). Differences in proportions of subgroups partly reflected variations in recruitment patterns. There were no differences in marital status and the subgroups were generally representative of the cohort described for the National study.

### Compliance with self-assessments

Of the eligible sample, 91% provided questionnaire data at 1 month and 79% at 12 months: 75% provided data on all three occasions. Participants who failed to provide follow-up data were significantly younger in age (mean age 32.3 *vs* 41.3 years; *P*<0.001) and comprised more individuals who perceived themselves to be at population risk level (43 *vs* 37%; *P*<0.411).

### Risk perception and change in relative risk accuracy

Using broad criteria for risk accuracy (population risk/elevated risk), 63% of attendees perceived their relative risk of cancer in the appropriate category as judged by the geneticist. There were three times as many subjects underestimating (27%) as overestimating (9%). These proportions were nonsignificantly reduced at 1 month (21 and 8%, *P*<0.06; McNemar's test) and at 12 months follow-up (20 and 7%, *P*<0.581; McNemar's test). There were no significant variations between centres, as shown in Table 3

**Table 3:** Psychological distress (GHQ and CWS): mean and median scores and changes over time

	**Centre A**	**Centre B**	**Centre C**	**Centre D**	**Centre E**	**All centers**
**Assessment point**	**(*n*=31)**	**(*n*=29)**	**(*n*=33)**	**(*n*=34)**	**(*n*=35)**	**(*n*=162)**
**Perceived personal risk^a^**	**Risk perception: Underestimators/all at elevated risk: *n* (%)**					
Pre-counsel	4/23 (17)	13/31 (42)	11/31 (35)	9/31 (29)	12/28 (43)	49/144 (34)
1 month	4/23 (17)	9/31 (29)	9/31 (29)	10/31 (32)	5/28 (18)	37/113 (33)
12 months	5/23 (22)	7/31 (23)	9/31 (29)	9/31 (29)	6/22 (27)	36/109 (33)
						
**GHQ**	**Mean, Median**	**Mean, Median**	**Mean, Median**	**Mean, Median**	**Mean, Median**	**Mean, Median**
Pre-counsel	4.8, 3.0	3.0, 0	3.5, 2.0	2.8, 1.0	3.3,1.0	3.4, 1.5
1 month	3.4, 0	2.3, 0	3.5, 1.0	2.6, 0	3.0, 0	3.0, 0
12 months	3.5, 1.0	2.9, 3.0	4.8, 4.0	3.3, 1.0	2.6, 2.0	3.4, 1.0
12-month pre-counsel^b^ (*P* value)^c^	−1.29, 0 (*P*=0.203)	−0.3, 0 (*P*=0.983)	1.3, 0 (*P*=0.200)	0.5, 0 (*P*=0.492)	−0.7, 0 (*P*=0.639)	−0.2, 0 (*P*=0.995)
						
**CWS**	**(*n*=35)**	**(*n*=32)**	**(*n*=39)**	**(*n*=38)**	**(*n*=38)**	**(*n*=182)**
Pre-counsel	11.1, 10	12.0, 12	11.0, 10	12.6, 11	11.2,11	11.6, 11
1 month	11.2, 10	11.1, 11	10.6, 10	11.1, 10	10.6, 10	10.9, 10
12 month	10.8, 10	11.0, 10	10.5, 10	11.4, 11	10.2, 10	10.8, 10
12-month pre-counsel^b^ (*P*-value)	−0.3, 0 (*P*=0.375)	−1.0, −1 (*P*=0.191)	−0.5, 0 (*P*=0.107)	−1.3, −1.5 (*P*=0.005)	−1.0, −1 (*P*=0.012)	−0.8, −1 (*P*<0.001)

a The ‘mean change’ and ‘median change’ for GHQ summary scores and ‘mean change’ and ‘median change’ for CWS summary scores are shown.

b Sample excludes subjects deemed by the genetics clinician to be at population risk.

c Wilcoxon signed rank test: Shown are ‘mean change’ and ‘median change’ for GHQ summary scores and ‘mean change’ and ‘median change’ for CWS summary scores.

.

### Psychological distress (GHQ)

There was no difference between the centres in GHQ mean scores at baseline or at either of the follow-up assessments (see Table 3) and no significant change over time for any of the centres. The prevalence of GHQ ‘probable cases’ for the full sample pre-counselling was 13%. By centre, this rate ranged from 6 to 23% pre-counselling, from 7 to 13% at 1-month follow-up and from 7 to 18% at 12-month follow-up, with no significant difference between the centres. There was no significant change in the prevalence rate over time (results for 162 subjects with full data).

### Cancer-specific distress (CWS)

At baseline, the mean cancer worry score for the overall sample was 11.6 (s.d. 3.0) and the median score was 11.0 (range 6, 22). Scores for the five centres are presented in Table 3, showing no significant differences between centres at any time point. A small but statistically significant reduction in cancer worry from baseline to the 12-month follow-up was observed only for centres D and E. However, combining the results for all the five centres, the mean/median change in cancer worry scores between baseline and 12 months was statistically significant (*P*<0.001). Across centres, reductions in the mean cancer worry scores from baseline to 1 month and from baseline to 12 months were similar for individuals who initially underestimated their risk (−0.3, −0.9) and for those who initially overestimated (−0.4, −1).

### Health care behaviour

Results are considered for women with a breast cancer risk, who formed the majority of attendees. Of 174 women with a breast cancer family history, 148 (85%) had ever had a breast examination, 40% in the 6 months before attendance at the CGS and 51% at the clinic. In all, 104 (60%) of these women had ever had a mammogram, 20% in the 6 months prior and 24% at the clinic visit. Results for 103 women deemed at increased risk of breast cancer, as judged by the clinicians, were comparable (see Table 4

**Table 4:** Health care behaviour: reported use of cancer screening tests

**Tests carried out on subjects with an increased risk (*n* (%))**					
	**Ever**	**In 6 months prior**	**At clinic**	**In 1 month since**	**In 12 months since**
*Increased breast cancer risk (n*=*103)*					
Breast exam	90 (87%)	47 (46%)	53 (51%)	4 (4%)	25 (24%)
Mammogram	61 (59%)	20 (19%)	22 (21%)	5 (5%)	27 (26%)
Breast U/S	32 (31%)	11 (11%)	N/K	0 (0%)	6 (6%)
CA125	27 (26%)	10 (10%)	N/K	5 (5%)	15 (15%)
Ovarian other	21 (20%)	2 (2%)	N/K	1 (1%)	5 (5%)
					
*Increased bowel cancer risk (n*=*28)*					
CA125^a^	1 (5%)	0 (0%)	N/K	0 (0%)	0 (0%)
Ovarian other^a^	4 (19%)	1 (4%)	N/K	0 (0%)	1 (5%)
Colonoscopy	15 (54%)	10 (36%)	N/K	1 (4%)	11 (39%)

a Women only (*n*=21).

). There were no differences observed between centres.

The proportion of women at elevated risk who were screened or examined within a year of the cancer genetics consultation varied significantly between centres, with between 54 and 100% receiving mammography (*P*<0.001) and the same range having a clinical breast examination (*P*=0.003) (data not shown). Two centres with low rates of reported access to mammography provided breast examination for only half of the women at risk, but it is not known whether this was routinely offered. Of note is the finding that 24% women at increased risk, and aged 35–49 years (the commonly recommended age cutoff for starting mammography), did not have a mammogram in the 6 months prior to or 12 months following the genetics consultation (see Table 5

**Table 5:** Reported use of mammography and clinical breast examination for female study participants, by age

**Examinations for women at elevated risk of breast cancer, by age^a^ (all time points included)**			
	**Mammography**	**Clinical breast exam**	**Clinical breast exam or mammography**
Age<35	5/37 (14%)	31/37 (84%)	32/37 (86%)
Age 35–49	38/50 (76%)	42/50 (84%)	45/50 (90%)
Age 50–65	10/15 (67%)	12/15 (80%)	12/15 (80%)
All	54/103 (52%)	86/103 (83%)	90 /103 (87%)

a Only one woman aged over 65 years.

); one in six of these women did not have a breast examination. Overall, 87% women at any age had one or other form of surveillance over this time period.

Numbers of individuals eligible for other cancer-screening tests were too few in number for comparisons between the centres, but, overall, only 54% individuals at risk for bowel cancer had ever had a screening test, of whom 36% had already undergone screened in the 6 months prior to attendance.

Breast self-examination habits were assessed at 1 and 12 months follow-up. Results showed no significant variation between centres (*P*=0.6, data not shown). A modest rate (40%) of usage of the frequently recommended monthly breast checking was reported overall, which remained stable over time, irrespective of the type of cancer risk. Women with a breast cancer family history were more likely to be excessive checkers (>once a week) than those with other cancer risks, both at 1-month follow-up (11 *vs* 0%) and at 12 months (9 *vs* 3%) (data not shown). Fewer women with a breast cancer history reported never checking compared with those with other cancer family histories (5 *vs* 21% at 1 month and 10 *vs* 18% at 12 months).

Use of clinical breast examination assessed at 12 months follow-up varied between 67 and 100% overall, across centres. Rates for women deemed at increased risk are shown in Table 5 and were uniformly high.

### Use of health care resources

Female participants were also asked about the use of specific health care resources, both before and following the genetic consultation. Numbers using such resources were too small to analyse by centre; therefore data are combined. Most counsellees did not have any contacts with the health service in the month following their consultation. Those returning to the CGS did so for a variety of appropriate reasons (data not shown). The likelihood of having a primary care appointment during the month varied by risk level (population risk 11%, moderate risk 19%, high risk 34%), but the likelihood of a hospital appointment did not. Table 6

**Table 6:** Health care visits since first cancer genetics consultation

**Health care resource**	**In following 1 month**		**In following 12 months**	
*Women with increased breast cancer risk (n*=*105)*				
Revisit CGS	10	10%	20	19%
Other hospital visit	20	19%	56	53%
GP surgery	36	34%	75	71%
Well-woman clinic	4	4%	8	8%
Family-planning clinic	2	2%	4	4%
Private health check	0	0%	4	4%

*Women with increased bowel cancer risk (n*=*22)*				
Revisit CGS	1	6%	4	24%
Other hospital visit	3	18%	10	59%
GP surgery	8	47%	11	65%
Well-woman clinic	0	0%	1	6%
Family-planning clinic	1	6%	2	12%
Private health check	0	0%	0	0%

shows that, for women with a breast or bowel cancer risk, 53 and 59%, respectively, made hospital visits and 71 and 65% had GP consultations in the year following the genetics consultation.

A minority of subjects (30.13%) changed their lifestyles since the consultation. In all, 12 (10 women and two men) adopted a healthier diet (more fruit and vegetables and less fat) and seven reduced weight. One stopped smoking and three reduced their alcohol intake. Numbers were too small for comparison by centre.

### Attendees' satisfaction with the CGS

Across all centres, satisfaction was reported as high at 1 and 12 months follow-up for all 14 aspects of service activity. The areas associated with greatest dissatisfaction are shown in Table 7

**Table 7:** Attendees reported satisfaction with overall service and with communication: comparison by centre

	**CentreA**	**Centre B**	**Centre C**	**Centre D**	**Centre E**	**All centres**
**Satisfaction item**	**% less than moderately satisfied**					
Plans for future screening	16	13	17	13	15	15
Risk management advice	17	3	26	7	30	17^a^
Wait for first appointment	11	6	22	22	29	18
Having cancer test/X-ray	12	25	41	13	3	16^a^
Info. on genetic test	13	13	41	15	23	20^a^
Lifestyle advice	23	14	39	9	41	27^a^
Availability of genetic test	22	30	39	18	42	30
Information re. prevention	20	22	40	17	45	30
						
*Fallowfield Satisfaction Scale – Mean (SD) summary score*						
1 month	62.1 (8.6)	64.2 (4.7)	57.8 (8.3)	63.4 (5.5)	59.5 (8.9)	61.3 (7.7)^b^
12 months	62.2 (7.3)	63.1 (5.6)	58.7 (8.6)	59.2 (10.1)	59.9 (8.0)	62.1 (8.3)^c^

a Significant at 5%. None were significant at 1%.

b Satisfaction with initial CGS consultation (*N*=176): *P*=0.02; Kruskal–Wallis test.

c Satisfaction with initial/most recent CGS consultation (*N*=171; 138 had not returned to the CGS): *P*=0.08; Kruskal–Wallis test.

, showing that 30% were dissatisfied with the availability of genetic testing and with information about cancer prevention strategies. Comparison of centres showed significant differences in dissatisfaction for risk management advice, having a cancer test/screening X-ray, information on genetic testing and lifestyle advice. The median waiting time for the first appointment varied significantly between centres from 19 to 31 weeks (*P*<0.001), but was associated with dissatisfaction for only 18% respondents overall, and this did not vary significantly between the centres. There was very little difference in satisfaction ratings at the two follow-up points, except for satisfaction with information given about the centre, which changed from 79 to 91% (data not shown).

### Satisfaction with communication

Satisfaction with the genetic consultation focused more specifically on communication and clinician style. Summary scores ranged between 57.0 and 64.2, with little change over the length of follow-up. There were significant differences between centres (see Table 7), with three items contributing to this variance: waiting too long to be seen at the clinic, not being told what the counsellee wanted to know and the doctor not seeming sympathetic (data not shown). No one centre was linked to dissatisfaction on all three items.

### Referring doctors' satisfaction

Questionnaires were sent to 198 doctors who had referred 238 participating subjects (range 1–10). Forms were returned by 116 (58.5%) doctors (67 GPs, 34 clinicians): three doctors were excluded as their role was integral to the genetics service and 79 (40%) doctors failed to reply. Reasons for noncompliance, where known, were: doctor or patient had left the practice (*n*=10); doctor had not made the referral himself (*n*=4); doctor too busy (*n*=2).

As shown in Table 8

**Table 8:** Referring doctors satisfaction with the cancer genetics service

	**Centre A**	**Centre B**	**Centre C**	**Centre D**	**Centre E**	**All centres**
**Satisfaction item**	**% Not at all satisfied/only a little satisfied**					
Referral criteria	5	16	28	8	20	18^a^
Waiting time for appointment	25	17	13	25	37	23
Feedback from CGC	0	0	0	4	6	2
Information about risk level	4	4	0	11	6	6
Information about screening	10	12	7	4	12	9
Prescribing information	22	0	11	9	15	11^a^
Information about risk management	13	5	8	4	24	11
Access to screening	5	13	14	15	12	12
Access to prevention	17	21	25	18	14	19

a Centres are significantly different at 5% (Kruskal–Wallis).

, over 90% of doctors responding were moderately or very satisfied with information about the patients' cancer risk, about cancer screening recommendations and with feedback in general from the genetics centre. However, 23% doctors (31% of GPs and 11% of specialists) were dissatisfied with the waiting time for an appointment, 18% (21% of GPs and 13% of specialists) were dissatisfied with referral criteria and 19% (20% GPs and 18% specialists) were dissatisfied with access to preventive treatment. There were significant differences in satisfaction between the centres for referral criteria and information about prescribing, such as use of hormone replacement therapy or the contraceptive pill. Specialists were more dissatisfied with information about prescribing than GPs (23 *vs* 6% GPs).

Centres associated with significant differences in doctors' dissatisfaction were different from those associated with significant differences in attendees' dissatisfaction, so that no particular service model appeared to be disadvantaged.

## DISCUSSION

This is the first direct comparison of psychosocial outcomes following genetic risk counselling for individuals with a family history of cancer in a UK-wide study of CGSs. In line with randomised comparisons carried out in Wales and South East Scotland, significant differences in psychosocial outcomes between the centres were small in number, with no discernible pattern in the centres concerned. Therefore, differences are unlikely to reflect a specific type of service configuration. The positive implication for the NHS is that service users will have similar psychosocial benefits despite wide variation in current service provision.

At first sight, the lack of differences may seem surprising, given evidence of wide variation in service structure, organisation and clinical workload (Wonderling *et al*, 2001). At the time the study was undertaken, CGSs were evolving to meet the model recommended by the Harper report (Working Group for the Chief Medical Officer, 1998), and other published information and guidelines (Murday, 1994; Ponder, 1994; Priority Areas Cancer Team, 1998; Eccles *et al*, 2000). It is possible that the information transferred in genetic risk counselling was broadly similar between centres, as a result of close collaboration within the genetics community, at a clinical and research level. We cannot be sure that differences in psychosocial outcomes do not exist for other CGSs throughout the UK, but the random selection of centres based on clinical activity levels in this study provides some basis for confidence in the findings, as extremes of variation were covered, and a consistent methodology applied. It is difficult to estimate the extent or impact of other risk counselling activity provided separately through breast clinics and community health settings, as no data are available.

This multicentre study confirms earlier published findings (Meiser and Halliday, 2002) that risk counselling is not harmful to general mental health, and achieves a modest effect on reducing cancer worry following a single consultation. Whether this benefit has an impact on risk management and health care behaviour cannot be shown by our findings, and warrants investigation. We were unable to show marked changes in risk perception, in contrast to published literature (Meiser and Halliday, 2002), but this is likely to reflect the variation in the type of cancer risks and the risk level in the study sample, which limited examination in detail. The impact of continued misperception of risk on health care behaviour and use of health resources remains to be determined.

The study also identified variations between centres that warrant attention. First, there were significant differences in the use of mammographic surveillance, which may partly reflect the lack of a national screening policy for high-risk women below the age of 50. Other factors could be a lack of access to mammography provision, poor communication at the interface between tertiary and primary care, or poor uptake/compliance with mammography, which were beyond the scope of this study. However, two initiatives should help to resolve this regional inequity and clarify the role of breast surveillance in this setting: these are the National Institute of Clinical Excellence (NICE) Clinical Guidelines for the classification and care of women at risk of familial breast cancer (NICE, May 2004; McIntosh *et al*, 2004), and the NHS Research and Development Health Technology Assessment evaluation of mammographic surveillance of women of age 40–49 with a family history.

The NICE guidance will also address areas of dissatisfaction raised by referring doctors, which warrant improvement. These concerned referral criteria and prescribing advice, while, for both doctors and attendees, there was perceived inadequacy of information about preventive options and access to genetic testing. Evidence is accumulating to support risk management interventions, such as risk-reducing mastectomy (Hartmann *et al*, 1999; Meijers-Heijboer *et al*, 2001), to help inform clinical decision-making, so that differences between centres observed here should gradually be eliminated. Clear criteria for referral to tertiary care and access to genetic testing will be provided in the NICE guidelines, reducing the potential for future dissatisfaction.

There were high levels of satisfaction across settings, in contrast to findings by Brain *et al* (2000), but significant differences in satisfaction between centres were observed for a small number of aspects of the consultation itself. This reflects the general need for identification of optimal risk communication strategies (Julian-Reynier *et al*, 2003) and communication training (Fallowfield *et al*, 2002). More obvious differences between centres, such as waiting time for the first appointment, did not discriminate levels of satisfaction between service users, but did cause dissatisfaction among referring doctors, and should be improved.

Differences between centres were not seen for primary care consultations or use of other health care resources following risk counselling, but the level of cancer risk did impact on resource use. Research initiatives have been undertaken to assist primary care teams in the identification of moderate and high-risk families who warrant further advice (Emery *et al*, 1999, 2000), so that individuals can be properly triaged for primary care support, cancer screening or cancer genetic referral. Use of health services in the year after the cancer risk consultation are difficult to interpret in the absence of a control group which did not receive genetic counselling, but hospital attendance rates did appear to increase, probably reflecting the need for cancer screening; this could be audited in light of the new service guidelines. Cancer genetics services can be an important means of optimising resource use through making appropriate recommendations to individuals most likely to benefit.

In contrast to other published work, we planned to evaluate a representative mix of individuals with different cancer risks, but all the five centres were dominated by referrals of women with a family history of breast cancer so that data are limited for smaller risk subgroups. For subjects with a risk of bowel cancer, the next largest subgroup, rates for colonoscopy usage were reported as lower than for screening mammography, but it is not possible to say whether this reflects lack of access or of uptake. Lower than expected rates of recruitment for colonoscopy have been reported elsewhere, and both discomfort and risks associated with the procedure were thought to be contributory factors (Eisinger *et al*, 2001). Other subgroups were too small for analysis. Women with a bowel cancer risk were less vigilant about breast self-examination and it is important that clear health care messages, such as the need to be ‘breast aware’, are provided for all women.

Strengths of the study design included the use of standardised assessments and the participation of randomly selected centres representing wide variation in service provision. Compliance with mailed questionnaires was generally good, but responses from referring doctors were lower. Some limitations are also acknowledged. The case mix and small sample quota from each centre, limited for logistical reasons, resulted in some lack of precision with which some outcomes could be assessed. Therefore, the study was designed to be descriptive rather than hypothesis testing. Gender and ethnic differences could not be evaluated and effort will be needed nationally to ensure appropriate representation of men and ethnic minorities in these clinics.

In conclusion, in the absence of differences in psychosocial outcomes, centres will need to think carefully about appropriate end points to evaluate the development of their particular service. There is now clear evidence of the information needs and perceived inadequacies of care as well as the level of mental health and satisfaction that can be achieved in this setting, that will form standards for future studies.
